# Modeling the within-host dynamics of HIV infection

**DOI:** 10.1186/1741-7007-11-96

**Published:** 2013-09-03

**Authors:** Alan S Perelson, Ruy M Ribeiro

**Affiliations:** 1 MS K710, Theoretical Biology and Biophysics, Los Alamos National Laboratory, Los Alamos, NM 87545, USA

## Abstract

The new field of viral dynamics, based on within-host modeling of viral infections, began with models of human immunodeficiency virus (HIV), but now includes many viral infections. Here we review developments in HIV modeling, emphasizing quantitative findings about HIV biology uncovered by studying acute infection, the response to drug therapy and the rate of generation of HIV variants that escape immune responses. We show how modeling has revealed many dynamical features of HIV infection and how it may provide insight into the ultimate cure for this infection.

## 

Since the discovery of HIV as the etiological agent of AIDS, numerous advances have been made in our understanding of the molecular biology, pathogenesis, and epidemiology of the virus, and the host immune response to it. Not least among these has been the knowledge obtained by mathematical analysis and within-host modeling of changes in viral load and T-cell counts after initiation of potent antiretroviral therapy in individual subjects. Indeed, modeling of the kinetics of HIV RNA under drug therapy has led to substantial insights into the dynamics and pathogenesis of HIV-1 [1-6] and the existence of multiple reservoirs that have made eradication of the virus difficult [7,8]. Through these analyses it has been possible to quantify the rapidity of HIV infection and replication, the rate of virion clearance, the lifespan of productively infected cells [1,2,4,5,9,10], and to predict the impact of treatment and the appearance of drug-resistant variants [11-13]. Other modeling efforts have helped clarify controversial issues relating to the mechanism of T-cell depletion in HIV infection [14] and motivated new experimental and clinical studies. More recent modeling studies have addressed issues such as immune escape and viral evolution, allowing a window into the quantification of the immune mechanisms operating in the setting of HIV infection.

Below we briefly review how quantitative data and modeling have contributed to the understanding of HIV biology.

## A model of HIV infection

**Figure 1:**
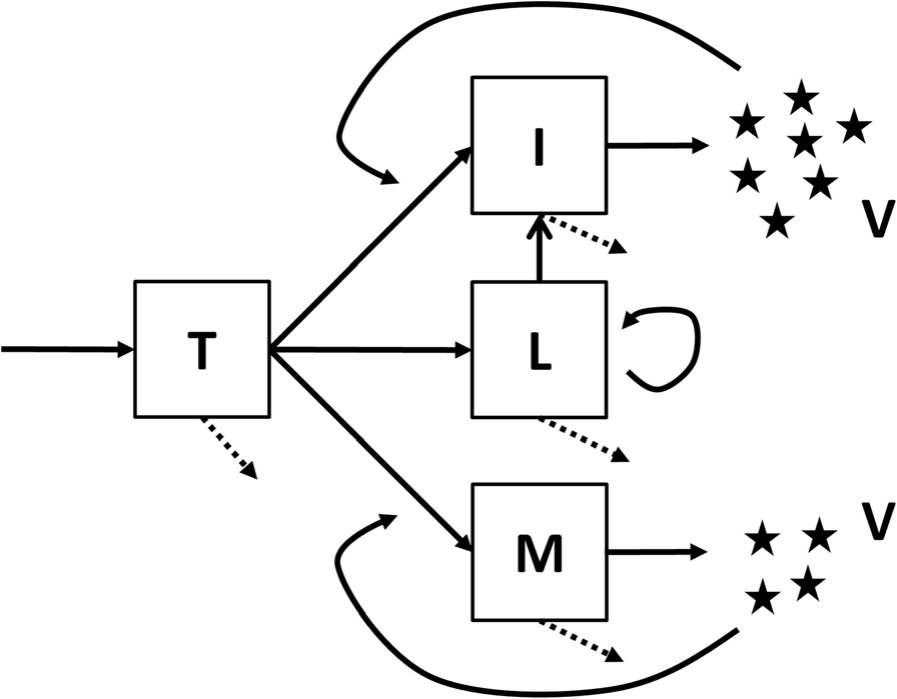
**Diagram of viral dynamics model.** Uninfected cells (*T*) can become infected by virus (*V*) to generate productively infected cells (*I*), long-lived infected cells (*M*) or latently infected cells (*L*). Latent infected cells may divide, sustaining this pool, which leaks to the productively infected class as latent cells are activated into cells producing virus. Dashed arrows indicate removal (death) of uninfected and infected cells, which occur at different rates. Equation (1) in the text considers only the uninfected cells, productively infected cells and virus.

In the simplest and earliest models of viral infection, only the key players in HIV infection were taken into account [1,2]. These models included uninfected target cells, *T*, infected cells, *I*, and free virus, *V* (Figure 1). Here target cells correspond mostly to CD4+ T cells expressing an appropriate co-receptor so as to be susceptible to infection. In this model, target cells are assumed to be produced at constant rate λ, to die at rate *d*_*T*_ per cell, and to be infected by free virus, according to a simple mass action infection term, that is, *βVT*. This generates productively infected cells, *I*, which are lost at rate *δ*, larger than *d*_*T*_, to reflect viral effects in shortening the infected cell lifespan. Finally, free viruses are produced by infected cells at constant rate *p* per cell, and are cleared from circulation at rate *c* per virus [15]. Thus, the differential equations describing this system are:

(1) $$\begin{array}{l} \frac{\mathrm{dT}}{\mathrm{dt}}=\lambda -d_{T}T-\mathrm{\beta VT}\\ \frac{\mathrm{dI}}{\mathrm{dt}}=\mathrm{\beta VT}-\mathrm{\delta I}\\ \frac{\mathrm{dV}}{\mathrm{dt}}=\mathrm{pI}-\mathrm{cV} \end{array}$$

This simple model was shown to be able to describe the kinetics of acute HIV infection [16,17] and the establishment of a steady-state - that is, a set-point - of viremia.

Only a small fraction of CD4+ T cells in the periphery become infected with HIV [18] and thus identifying the target cells in this model is not straightforward. However, the model is able to describe the kinetics of T-cell depletion in macaques infected with an X4-tropic virus, such as SHIV89.6P, where most T cells are target cells [19]. Despite its ability to fit data, this model may be too simple in that it does not include any explicit immune response (more on this below). Nonetheless, this simple model and generalizations that include long-lived infected cells and latently infected cells (Figure 1) have proven to be useful and have generated important insights into the biology of HIV [4,9,20].

## Modeling antiretroviral therapy

### Early decay

**Figure 2:**
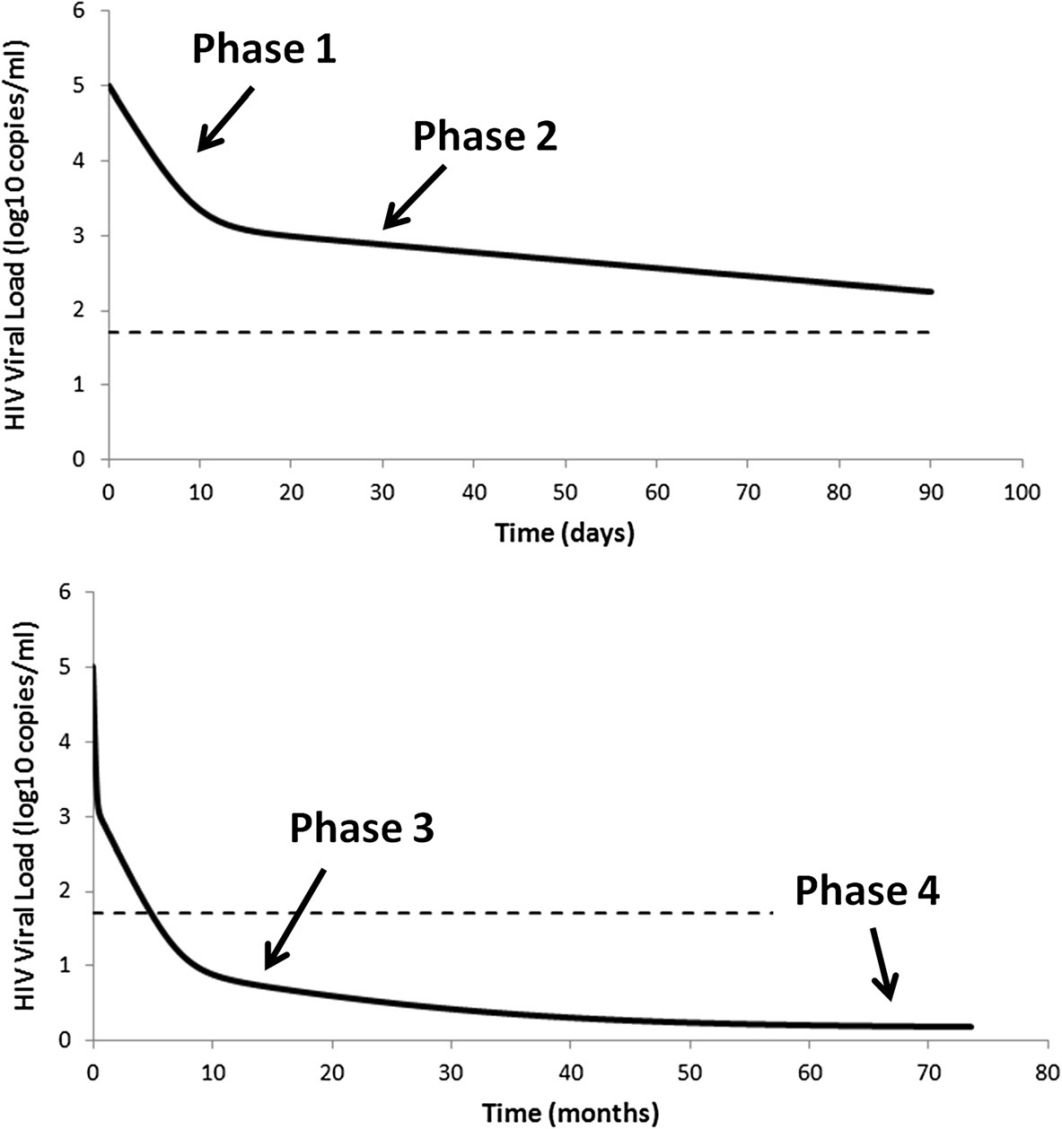
**Phases of viral decay under treatment.** When treatment is initiated, plasma viral load undergoes a multiphasic decay, with slower rates of viral loss as treatment progresses. One possible explanation is that there are various classes of infected cells (Figure 1) with different turnover rates. This phenomenon makes it very difficult to predict whether viral eradication is possible and how long it would take.

The effects of antiretroviral therapy can easily be included in equation (1), so as to analyze the dynamics of viral decline under different therapeutic regimes. For example, reverse transcriptase inhibitors can prevent the establishment of productive infection of a cell. To model this the infection term *βVT* in equation (1) is replaced by (1 - ϵ_RTI_)*βVT*, where ϵ_RTI_ is a number between 0 and 1 called the effectiveness of the reverse transcriptase inhibitor. Here ϵ_RT_ = 1 implies a 100% effective inhibitor. Protease inhibitors (PIs) prevent the maturation of HIV virions into infectious particles. To model PIs, the viral population is split into two populations, *V*_*I*_ and *V*_*NI*_, where *V*_*NI*_ represents immature non-infectious particles created by the action of the PI [4], and the viral equation in equation (1) is replaced by the two equations:

(2) $$\begin{array}{l} \frac{dV_{I}}{\mathrm{dt}}=\left(1-\epsilon_{\mathrm{PI}}\right)\mathrm{pI}-cV_{I}\\ \frac{dV_{\mathrm{NI}}}{\mathrm{dt}}=\epsilon_{\mathrm{PI}}\mathrm{pI}-cV_{\mathrm{NI}} \end{array}$$

where ϵ_PI_ is the effectiveness of the PI. This simple model has been used successfully to fit viral load data taken from individuals on antiretroviral therapy [2,4]. The model explained the viral decays seen over the first week or two of therapy, what we now call the first phase of viral decay (Figure 2). Because free virus is cleared very rapidly, with a half-life of about 45 minutes [21], the amount of virus measured in plasma after the first few hours of therapy reflects the production of virus by infected cells and as such the rate of decay of plasma viremia reflects the loss rate of productively infected cells when therapy is 100% effective. If therapy is less than 100% effective, then the observed decay rate is slower than the rate of loss of productively infected cells as some viral production continues. Thus, comparing the first-phase decay rates of various drug regimens allows one to compare their relative effectiveness [22] and using the most potent regimens that approach 100% effectiveness has led to the conclusion that productively infected cells live about one day after they start producing virus [23]. Recently, this concept of treatment effectiveness has been taken further by modeling the inhibitory potential of different drug combinations, at clinical concentrations, based on an extensive set of *in vitro* experiments for most of the current anti-HIV drugs [24,25].

### Second phase decay

If one follows the response to combination antiretroviral therapy (cART) for more than a week or two, one sees that the rapid first phase of decay of plasma viremia is followed by a slower second phase of decay (Figure 2). This second phase has been attributed to the existence of longer-lived productively infected cells, perhaps resting CD4+ T cells or cells of the macrophage/monocyte lineage [5]. Indeed, there is clear heterogeneity in the cell types that are infected by HIV and in the amount of virus produced by these cell types [26], consistent with the suggestion that some infected cells may live substantially longer than others. Nonetheless, some modelers have suggested other explanations for this second phase, such as the decline being driven by cytotoxic T lymphocytes (CTLs), which slows as the CTL response declines [27], or that infected cells have an age-dependent transactivation rate, which slows the generation of virus-producing cells [28].

Irrespective of the mechanism generating the second phase, with continued cART viral levels decline below the detection limit of clinical assays (50 HIV RNA copies/ml), and with these assays one cannot determine how long the second phase lasts.

### Third and fourth phase decays

Based on the rate of second phase decay, modeling suggested that 3 to 4 years of fully suppressive therapy could eliminate the cells responsible for second phase virus [5]. However, the use of single copy assays (SCAs), which allow one to detect as few as one HIV RNA/ml of plasma [29], has led to the identification of a third phase of decay with a half-life estimated as 39 weeks in one study [30] and 69 weeks in another [31] with overlapping and rather large 95% confidence intervals. Palmer *et al.*[30], as well as others [29,32], also suggested a fourth phase or constant level of viremia is attained after very long times on therapy (Figure 2). The existence of these later phases implies that it may not be possible to eliminate HIV with antiviral therapy alone. The sources of third and fourth phase viremia are controversial and may include leakage of bound virus from follicular dendritic cells (FDCs) [33-35], the release of virus from latently infected cells and ongoing viral replication.

### Latency

HIV incorporates into the host cell genome and can establish a latent form of HIV infection, involving a small fraction of resting memory CD4+ T cells that carry integrated viral genomes [18,36,37]. Longitudinal analysis suggested that this latent reservoir could persist in patients for as long as 60 years [7]. While latently infected cells do not produce virus in the resting state, they can do so upon activation [36]. This feature has been explored by modelers to explain the occasional viral ‘blips’ seen in patients who are otherwise well-suppressed [38-42], as well as the low levels of plasma viremia [30,41,43-46] detected with research assays that have lower limits of detection in the order of one HIV RNA/ml [29]. Since the latent pool is not depleted by this occasional activation, modeling suggested the possibility that latently infected cells undergo homeostatic proliferation without activation and thus maintain the latent reservoir for decades [41,45]. This hypothesis was later experimentally confirmed [47].

Modeling has also examined the role of early therapy in preventing the establishment of latency [48] and work is underway by both experimental and modeling groups examining the possibility of activating latently infected cells by therapies, such as the use of histone deacetylase inhibitors, to potentially generate a cure for HIV [49,50]. In assessing such new therapies, models for the activation of latently infected cells [5,41,46] may provide critical insights.

So far, we have described the ‘ideal’ scenario for antiretroviral therapy, where treatment is successful in driving the viral load below detection levels. However, viral load sometimes rebounds and new viral strains with mutations that confer resistance to the drugs used in the treatment protocol are observed.

## Drug resistance

When HIV infects a cell, the viral RNA genome is reverse transcribed into DNA. The reverse transcription process is error prone and results in mutation at an estimated rate of 3 × 10^-5^ per base per generation, with about two-thirds of these mutations being nucleotide substitutions [51]. Thus, when HIV with its almost 10 kb genome is reverse transcribed there is about a 20% chance that a base substitution occurs. In a chronically infected patient it has been estimated that about 10^8^ cells are infected each day, thus allowing all possible single and many double mutations to be explored each day [3,11]. Also, drugs may not penetrate all tissues and all cells with equal efficiency, and drug ‘sanctuaries’ may exist [52]. Thus, it is not surprising that resistance to antiretroviral drugs can develop relatively quickly, if combination therapy is not employed. This was explained by modeling within-host viral dynamics [12,13,53-58]. Moreover, several quantitative studies indicated that resistance is more likely to pre-exist before therapy is started than to appear *de novo* during the therapy, especially if therapy is strong enough to curtail replication [12,54,59]. Three factors contribute to the development of resistance, and indeed to the observed diversity of HIV: i) its high mutation rate, which is typical of an RNA virus, ii) its fast lifecycle, and iii) the long-term nature of HIV infection. These three factors combine to allow rapid viral evolution and the generation of high diversity.

More recently, studies have shown that some patients derive clinical benefit from continued therapy even when the virus is resistant to the drug protocol, as the resistant virus can be less fit than wild type [60].

Even though the introduction of antiretroviral treatment was a great success, and improved the quality of life of countless people, it has not become the panacea that was expected. For several reasons, including those alluded to above, treatment has not yet cured HIV. Thus, it has become clear that the development of a vaccine that prevents infection in the first place or ameliorates the course of disease is very important. To this end a better understanding of the clinical progression of HIV and the immune response against it is crucial. Here too, models have been playing a critical role as we discuss below.

## Viral dynamics in primary infection

HIV is enormously diverse [61]. Thus, it was very surprising to discover that approximately 80% of sexually transmitted infections are the result of a single transmitted/founder virus [62]. This discovery was a perfect example of extraordinary technical developments in assay capabilities pushing the state-of-the-art in modeling to analyze the results generated by those assays. In this case, single genome amplification (SGA) assays allowed an unprecedented look at the phylogenetic structure of HIV early post-infection. To analyze these data, a stochastic model of viral diversity generation was developed [63,64]. The results conclusively showed that the observed early genetic structure was most compatible with a single virus being transmitted or founding the complete viral population in a large fraction of heterosexual transmissions.

**Figure 3:**
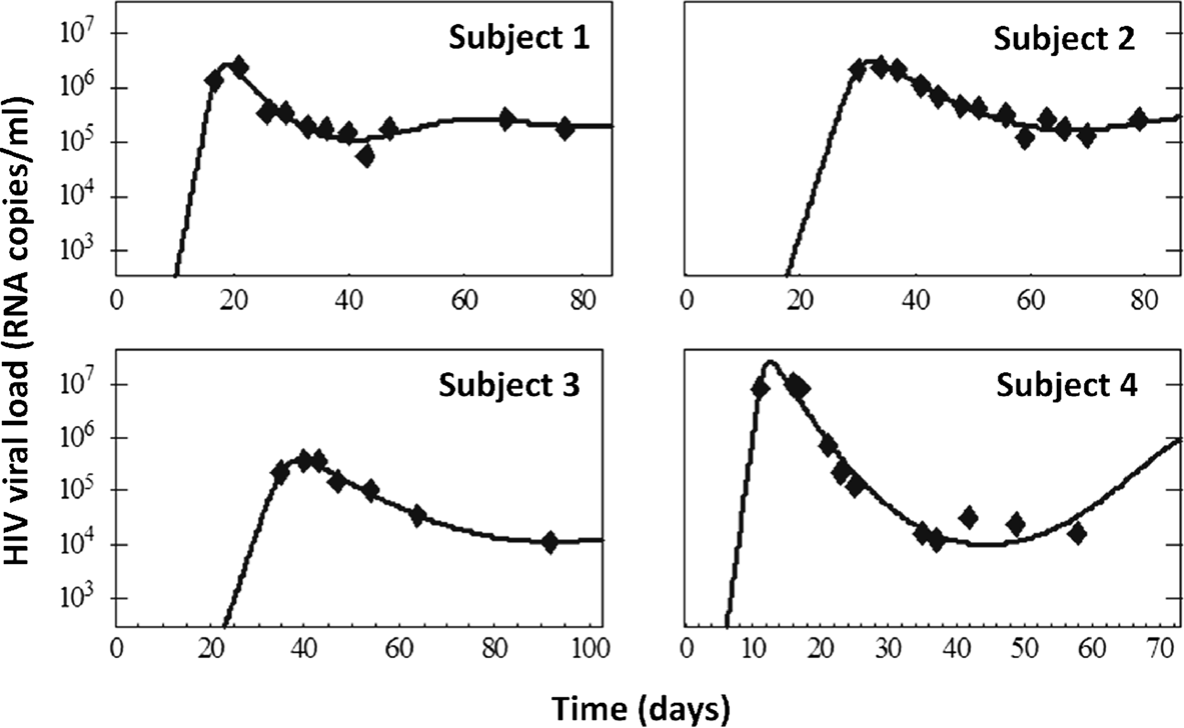
**Data fits in primary infection.** The basic model of HIV dynamics (equation (1)) provides a good description for the initial period of viral infection, from the time of infection through the initial peak in viral load and subsequent control and achievement of a quasi steady-state (data from [16]).

A number of other modeling questions are of interest in the setting of acute HIV infection. Very early on, it was realized that the simple viral dynamics model in equation (1) could explain the behavior of the virus during early infection [2,4,17]. The model reproduces the fast exponential rise of the virus, the achievement of a viral peak and the precipitous decline that ensues to a quasi-steady state level, termed the ‘set-point’ [16] (Figure 3). The exponential increase in viral load, which has been estimated to correspond to a doubling time of 0.65 days [65], has been used with models to determine the basic reproductive number, R_0_, for HIV [16,65,66] and SIV, a simian model for HIV infection [67]. The current best estimate of R_0_ for HIV is about 8, suggesting that each infected cell on average infects 8 others [65]. While models predict the set-point [16,68], no simple interpretation of what determines the large variation in set-point among individuals, at least three orders of magnitude [69-71], has been found, as the set-point viral load depends on all of the parameters in the basic model shown in equation (1) [16]. However, Bonhoeffer *et al.*[72] suggest that most of the variation in set-point is due to variation in the rate at which activated CD4+ T cells are produced.

Not every encounter with HIV/SIV results in infection; the transmission probability for HIV has been estimated as 10^-3^ to 10^-2^ per coital act [73]. This suggests that when low levels of virus are transmitted the infection may go extinct rather than take off. This possibility, as well as the potential efficacy of pre- and post-exposure prophylaxis, has been explored though the use of stochastic models of early infection [74,75]. These models suggest that extinction would most likely occur well before virus is detectable even with single copy assays. Thus, the low observed HIV-1 transmission rate may be a consequence of small numbers of virions being transmitted followed by frequent extinction.

Although the modeling studies mentioned so far, dealing with a variety of different processes in HIV infection, have been quite successful in advancing our understanding of HIV biology, a puzzling aspect remains: these models do not necessarily include an explicit immune response.

## Immune responses during HIV infection

The basic model given by equation (1), lacks an explicit representation of the immune response and hence has been called a target-cell limited model [76]. Despite this deficit, the model fits viral kinetic data obtained both during natural infection (Figure 3) and while patients are on therapy. However, immune responses against HIV, at both the cellular and humoral level, can be detected and there are considerable data indicating a role for CD8+ T-cell responses during HIV infection, particularly in people called elite controllers [77-79]. Further, depleting CD8+ T cells during acute SIV infection is associated with SIV remaining at high levels rather than reaching a distinct peak and then rapidly falling [80]. Also, HIV-1 tends to start accumulating CTL escape mutations around the time of the peak viremia, supporting the notion that CD8+ T cells play an important role in controlling early viremia [81]. On the other hand, increasing the initial number of HIV-specific CD8+ T cells, by vaccinating macaques prior to infection, did not change the growth rate or decay rate of virus from its peak during primary infection, suggesting a limited role of CD8+ T cells [82,83]. How to reconcile these various observations and account for the initial immune response in models of acute infection is still a subject of research and debate [84,85].

While many models have included CD8 responses [68,86], more than we can review here, they tend to lack comparisons with experimental data leaving the field without good estimates for the parameters that govern CTL effects. An interesting example of these types of models was developed to analyze experiments of SIV in macaques where early drug treatment led to control of the virus in the long term (akin to ‘elite controllers’) [87,88]. In these models, the interactions between virus, CD4+ T cells and CD8+ T cells were considered [89], as HIV-induced depletion of CD4+ T cells may affect one’s ability to mount CD8+ T-cell responses [90].

Modeling antibody responses to HIV is still an undeveloped area, although some work involving data interpretation has been done [91,92]. Interestingly, in modeling acute SIV infection an improvement in fit to the measured viral loads was attained by allowing the viral infectivity, β in equation (1), to decrease with time, possibly reflecting the effects of antibody that builds up over time in reducing viral infectivity [93]. This effect was suggested by the work of Ma *et al.*[94] that showed mixing set-point plasma with acute-phase plasma decreased the infectivity of the acute-phase plasma.

**Figure 4:**
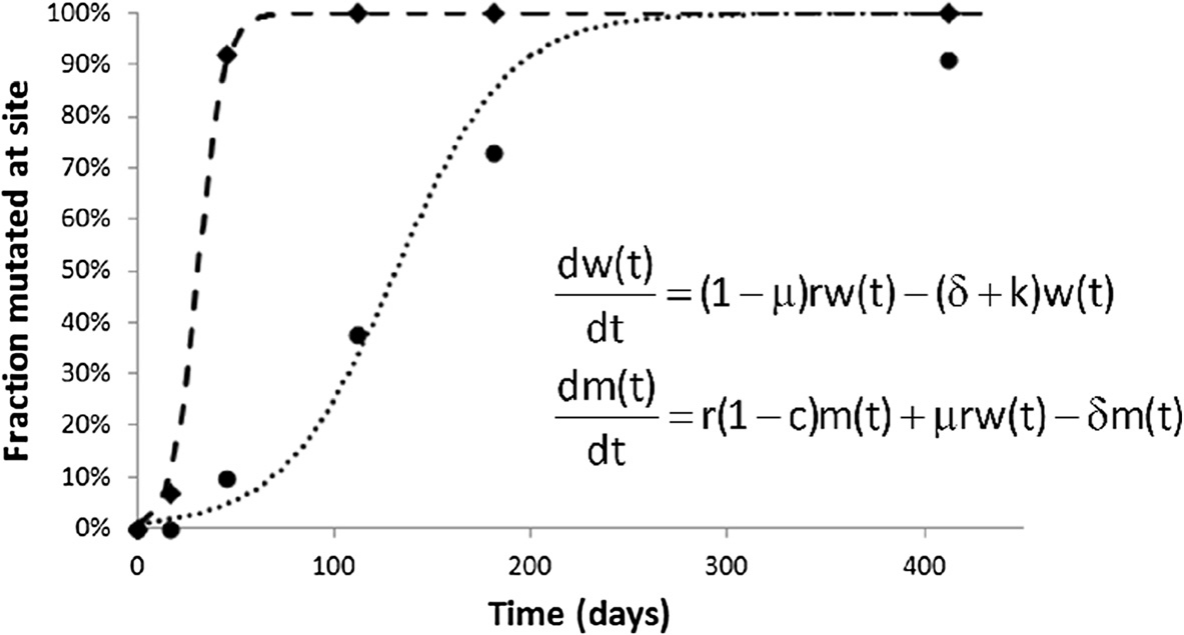
**Immune escape at cytotoxic T lymphocyte epitopes.** Schematic representation of the takeover of the viral population by virus mutated at a particular CTL epitope escaping the host immune response. Two different epitopes (diamond and circle symbols) are represented with different rates of population turnover. The equations shown represent the dynamics of wild-type (*w*) and mutated virus (*m*) as they replicate with different rates (*r* and *r*(1 - *c*)), where *c* is the ‘cost’ of escape, and the wild-type virus mutates to escape virus at rate μ (note that for simplicity we do not represent the backward mutation). CTL kills wild-type virus at rate *k*, but does not affect the escape virus.

One method of trying to estimate the strength of both humoral and cell-mediated immune responses is to determine how fast the virus can escape from these responses [81,92]. The basic idea, which we discuss in terms of cell-mediated responses, is that cells infected by wild-type virus should be susceptible to both viral cytopathic effects and immune-mediated killing, say by CTL responses, whereas a ‘CTL escape variant’ would only be susceptible to viral cytopathic effects. Following this approach, the basic model of viral dynamics in equation (1) has been generalized by a number of modelers to include both wild-type and escape mutant virus and cells infected by these two classes of virus [95-99]. Assuming that the concentration of virus is proportional to the density of cells that produce that virus, these models can be simplified to the two equations shown in Figure 4 for wild type, *w*, and mutant, *m*, virus [99]. These equations can then be solved to yield the frequency of escape (mutant) virus as a function of time since the start of infection [95,99]. As one might intuit, this frequency increases at a rate dependent on how fast the escape mutant grows relative to the wild type. This rate, called the escape rate, increases proportional to the rate of CTL-mediated killing of the wild type, *k*, and decreases with the fitness cost of escape, *c*. Thus, the fastest escapes would occur when the CTL pressure, *k*, is high, and the cost of escape, *c*, is low; whereas when there is weak CTL pressure and a high cost of escape, the escape rate should be low [95-100].

While models with only two viral species are easy to analyze, simulation models have looked at much more complex situations in which there are multiple escapes [101]. Further, recent data using single genome amplification and sequencing shows that HIV can escape at multiple T-cell epitopes [81,102]. Thus, models are being developed that consider the entire HIV genotype and not simply escape at single epitopes that are treated independently [103].

Some studies quantified the efficiency of cytotoxic T lymphocytes in killing infected cells based on the rate of escape of viral variants at specific epitopes. These results were used to compare the effect of immune pressure in macaques infected with SIV with that of humans [104]. However, precise estimates of escape rates were hampered by a lack of frequent sampling. Recently, a large effort has been expended by the Center for HIV AIDS Vaccine Immunology (CHAVI) to elucidate the early immune response during primary infection. Identifying subjects with a single transmitted/founder sequence and then following the evolution of that sequence in time using either deep sequencing [105] or single genome amplification techniques has made it possible to study the dynamics of the emergence of both antibody and CTL escape variants in some detail [81,92,102]. While early results suggested CTL response against a single epitope only provides a modest amount of pressure [104], later work using much more frequent patient sampling showed that the CTL response against a single epitope could account for as much as 35% of the killing [81].

Balamurali *et al.*[106] measured the post-peak maximal viral decay rates of wild-type and escape mutant virus in macaques and found them to be the same, suggesting that CD8+ T cells may act by non-cytolytic mechanisms. We [107] and others [108] tried to address this issue directly by modeling data on the decay rate of virus during cART in the presence and absence of CD8+ T cells [107]. Our results were consistent with CD8+ T cells mainly killing cells before they began producing virus or with CD8s mainly having a non-cytolytic effect, but a later analysis showed a small cytolytic effect on productively infected cells could not be ruled out [109]. Experiments that determine the rate of viral decay during cART only provide information about the fate of productively infected cells. Thus, other modeling studies have examined the possibility that CTLs act by killing cells before they become productively infected [110,111]. Studies of escape have also been used in other imaginative ways - for example, to estimate the turnover of integrated DNA in resting CD4+ T cells, which is one of the blocks in the elimination of infection [112].

As AIDS is characterized by a loss of CD4+ T cells, much modeling work has focused on quantifying T-cell turnover during HIV infection using direct labeling as well as cell markers such as T-cell receptor excision circles. Much of this modeling literature has recently been reviewed [113] and thus will not be discussed here.

## Future perspective

Modeling HIV dynamics has been a rich area of study that, we believe, has spearheaded the wider field of modeling in viral and immune system dynamics. Indeed, there is much more research in HIV modeling than we could possibly cover here, but we hope to have given a representative flavor of the most innovative studies.

Looking into the future, there are several areas where we believe modeling can still make important contributions. Clearly, as more quantitative data on the immune response, both cellular and humoral, become available, it will be important to include these in the mechanistic models of HIV infection. This will be especially important to explain the events during primary infection, and in the analysis of vaccine trial data in humans. These models should be able to describe not just the time evolution of the viral load, as do current models, but also the kinetics of the immune response and its effect on virus and infected cells. Another area of interest to modelers is the potential effect of cell-to-cell transmission of HIV-1 [114-119]. If a sizeable proportion of infections *in vivo* occur this way, the virus may be able to avoid antibody effects, which mostly affect free virus, and thus evade the effect of the humoral immune response. Also, the transfer of multiple viral genomes by cell-to-cell transmission can reduce the efficiency of cART [120]. Finally, as efforts intensify on finding therapies to activate latent cells, models quantifying the effects of such therapies and the balance of re-activation and new infections could help inform best protocols for the clinic.

Overall, we believe that two of the most pressing questions in the HIV field are why primary infection invariably leads to chronicity, and why chronic infection leads to persistence of the virus, even in the face of very potent and durable therapy. It is unquestionable that future modeling studies of novel datasets will help us to understand both of these questions, proposing different mechanisms and hypotheses for these observations. In so doing, we might uncover new intervention strategies to help prevent or eradicate infection. The idea of curing HIV infection either, by viral eradication or functionally curing the infection by controlling it, is gaining traction [121,122]. Modeling will surely play a role in these endeavors, and in this way modeling will have come full circle from early insights into viral biology to demonstrating that cure is possible.

## Conclusion

Mathematical analysis of HIV-1 viral dynamics and immune responses has led to a number of important insights about the dynamics and pathogenesis of HIV infection. Modeling plasma virus decay under therapy demonstrated the fast turnover of virus, explaining the potential for generation of mutants and the development of drug resistance. This early work paved the way for many collaborations between clinicians and modelers to understand the nature of the multiphasic viral decay observed in treated patients, the initial expansion of virus upon infection, the turnover of CD4+ and CD8+ T cells, the probability of single transmitted/founder viruses and many others. It is fair to say that most, if not all, of these insights would not have been possible without close interdisciplinary collaborations allowing quantitative modeling.
